# Complete Atlantooccipital Assimilation with Basilar Invagination and Atlantoaxial Subluxation Treated Non-Surgically: A Case Report

**DOI:** 10.7759/cureus.1327

**Published:** 2017-06-09

**Authors:** Ali J Electricwala, Amita Harsule, Vishwajeet Chavan, Jaffer T Electricwala

**Affiliations:** 1 Department of Orthopaedics, Electricwala Hospital and Clinic; 2 Radiologist, Ruby Hall Clinic, Ruby Hall Clinic, Wanworie, Pune, Maharashtra, India; 3 Orthopaedics, Orthocarespecialtyclinic, Satara Road, Pune

**Keywords:** complete atlantooccipital assimilation, basilar invagination, atlantoaxial subluxation, predisposing anterior cord compression, treated non-surgically

## Abstract

Atlantooccipital assimilation is a partial or complete congenital fusion between the atlas and the base of the occiput. Most patients with atlas assimilation are asymptomatic, but some may present with neurological problems such as myelopathy. We present the case of a 37-year-old woman who presented with neck and occipital pain, episodic neck stiffness, and dizziness. Medical imaging revealed complete atlantooccipital assimilation associated with basilar invagination, atlantoaxial subluxation, and predisposing anterior spinal cord compression. The patient was treated non-operatively with medications, cervical interferential therapy, and a rigid cervical orthosis.

## Introduction

The craniovertebral junction (CVJ) comprises of the occiput, atlas, and axis [1]. Occiput anomalies consist of condylus tertius, condylar hypoplasia, basiocciput hypoplasia, and atlantooccipital assimilation. Atlantooccipital assimilation is a partial or complete congenital fusion between the atlas and the base of the occiput [2]. This occurs due to the failure of segmentation between the fourth occipital sclerotome and the first spinal sclerotome [3]. The term atlantooccipital assimilation has been used interchangeably with occipitalisation and atlantooccipital fusion. This condition may lead to chronic atlantoaxial instability or basilar invagination [4]. The symptoms may vary depending on the area of spinal cord impingement. In the anterior cord impingement, pyramidal signs and symptoms predominate. In the posterior impingement, posterior column signs and symptoms predominate. In this case, our patient presented with neck and occipital pain, radiating to both shoulders. Medical imaging revealed a complete fusion of the lateral masses of the atlas with the occipital condyles, in association with basilar invagination, atlantoaxial subluxation, and predisposing anterior spinal cord compression.

## Case presentation

**Figure 1:**
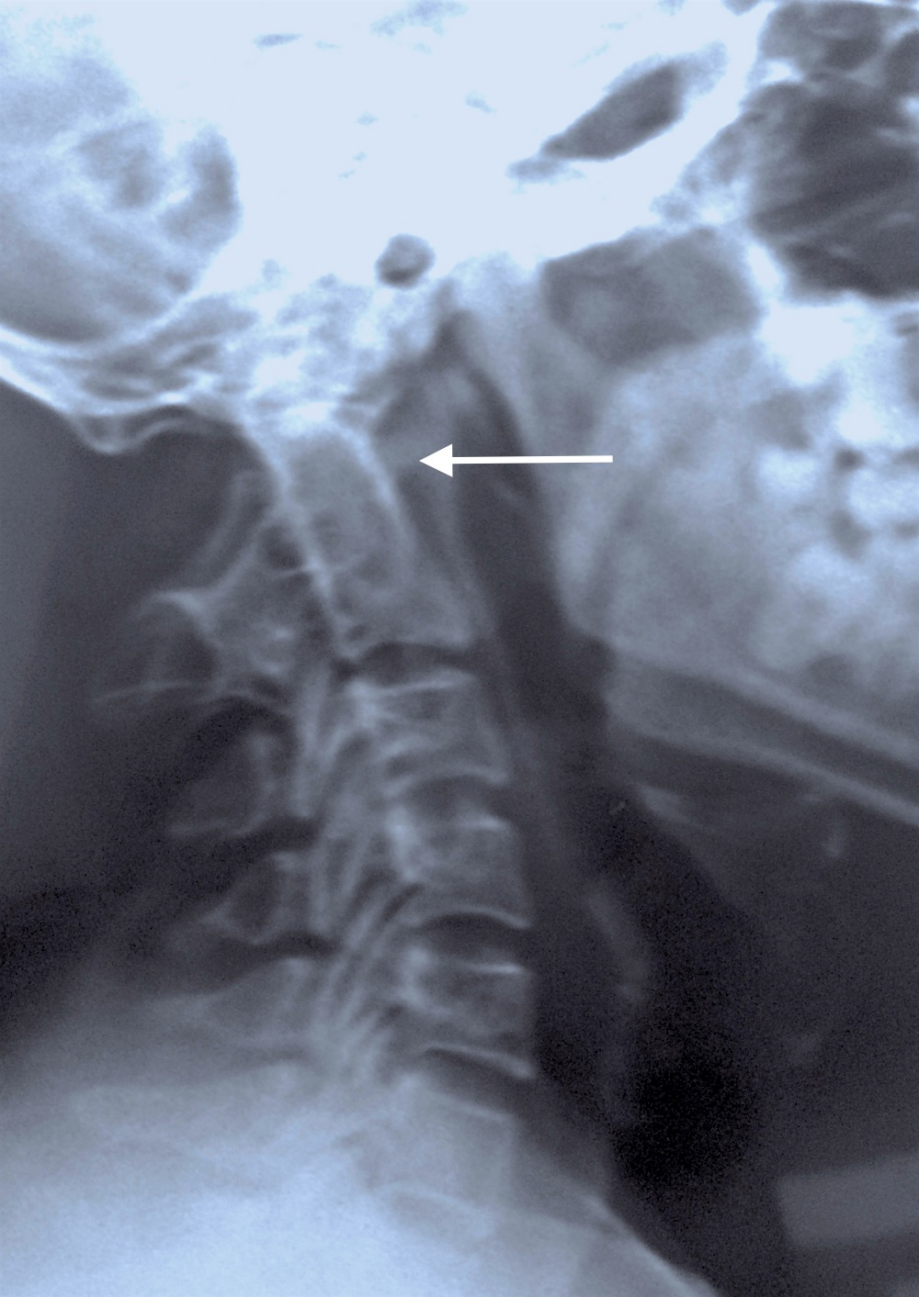
Lateral radiograph of the cervical spine falsely interpreted as "congenital absence of atlas" (arrow)

A 37-year-old lady presented to our clinic with dull, aching pain in the neck and occiput since a fortnight, which began after lifting a heavy object. The pain was sudden in onset, dull-aching in character and was associated with episodic neck stiffness. The pain radiated to both shoulders. A general examination of the patient was unremarkable except for a low hairline and a short neck. There were no signs of anterior (negative Babinski and Hoffman signs) or posterior spinal cord compression (normal fine touch, proprioception, sterognosis, and vibration sense). Her cranial nerve function was normal. Her sensory and motor examinations were normal in both upper and lower extremities. Deep tendon reflexes were physiological and symmetrical. Ambulation was normal. A routine radiograph was obtained which was initially falsely interpreted as “complete absence of atlas” (Figure 1). 

**Figure 2:**
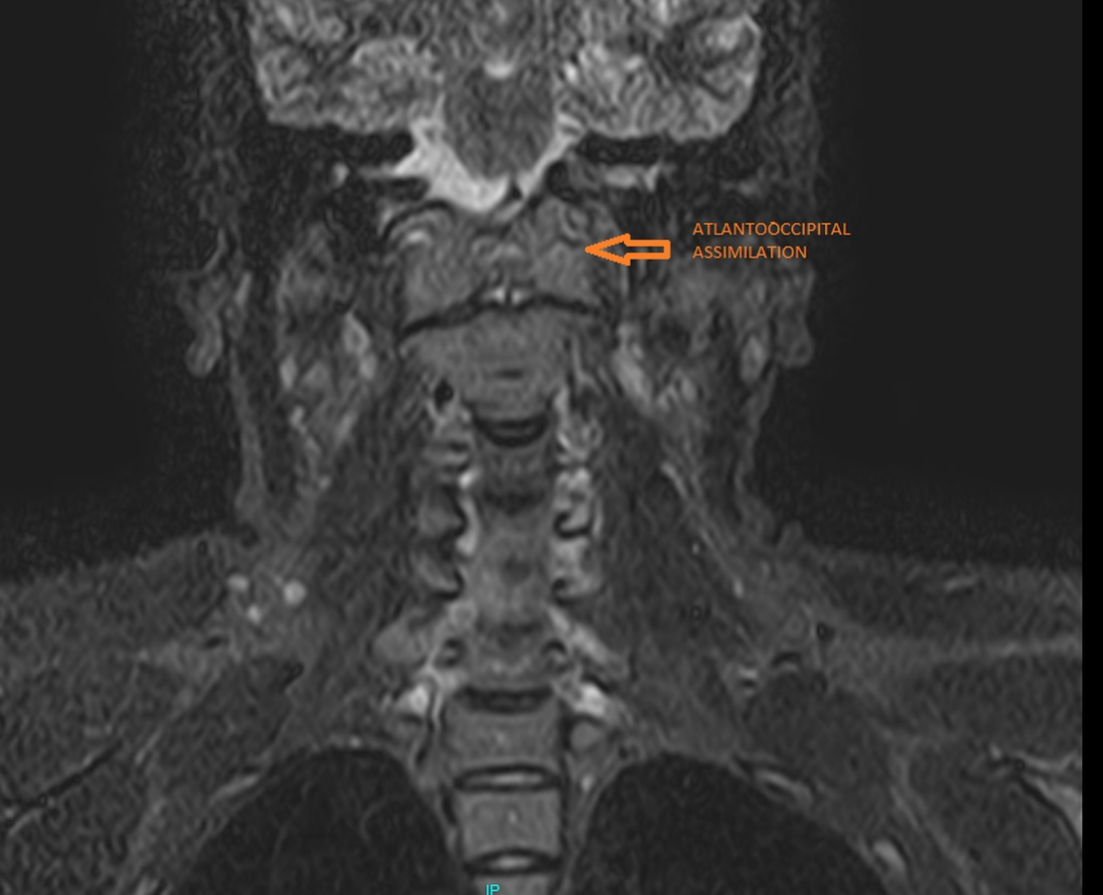
Coronal short tau inversion recovery (STIR) magnetic resonance imaging scan showing complete atlantooccipital assimilation (arrow)

**Figure 3:**
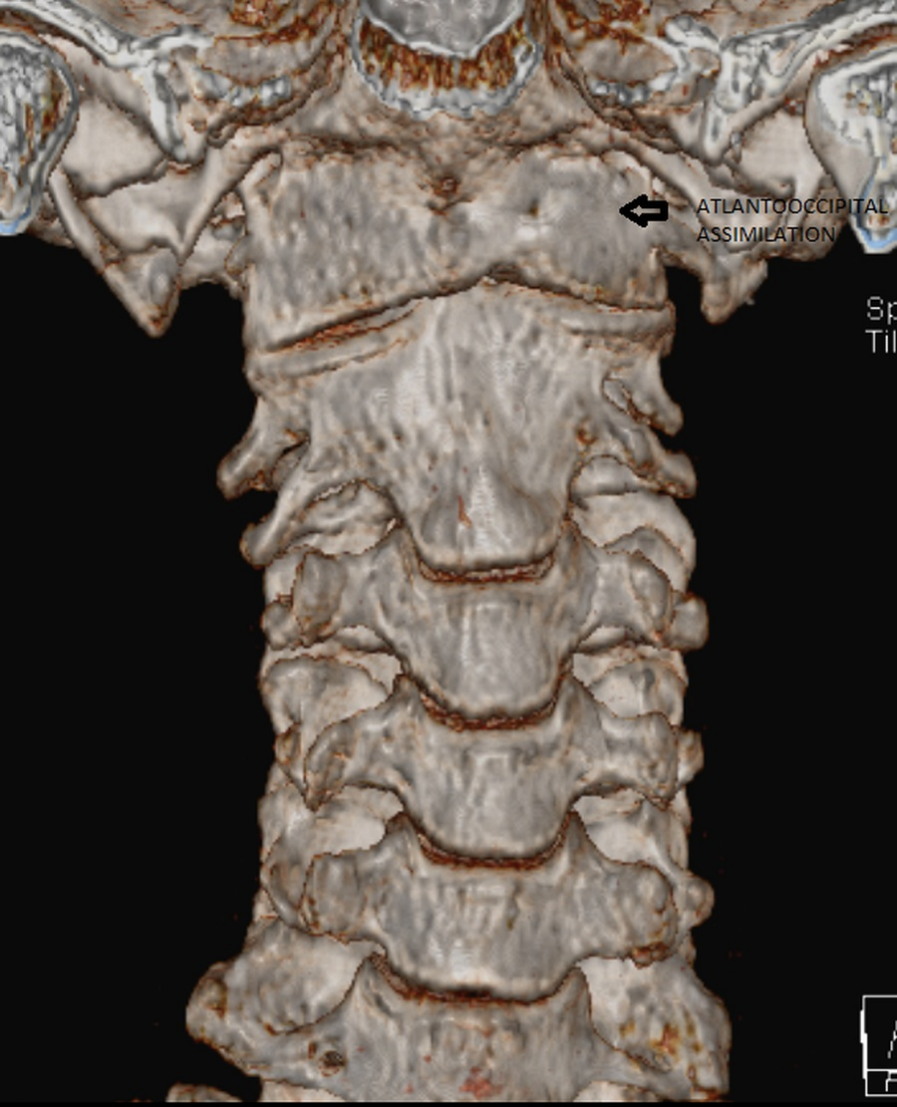
3D computed tomography scan showing complete atlantooccipital assimilation (arrow)

**Figure 4:**
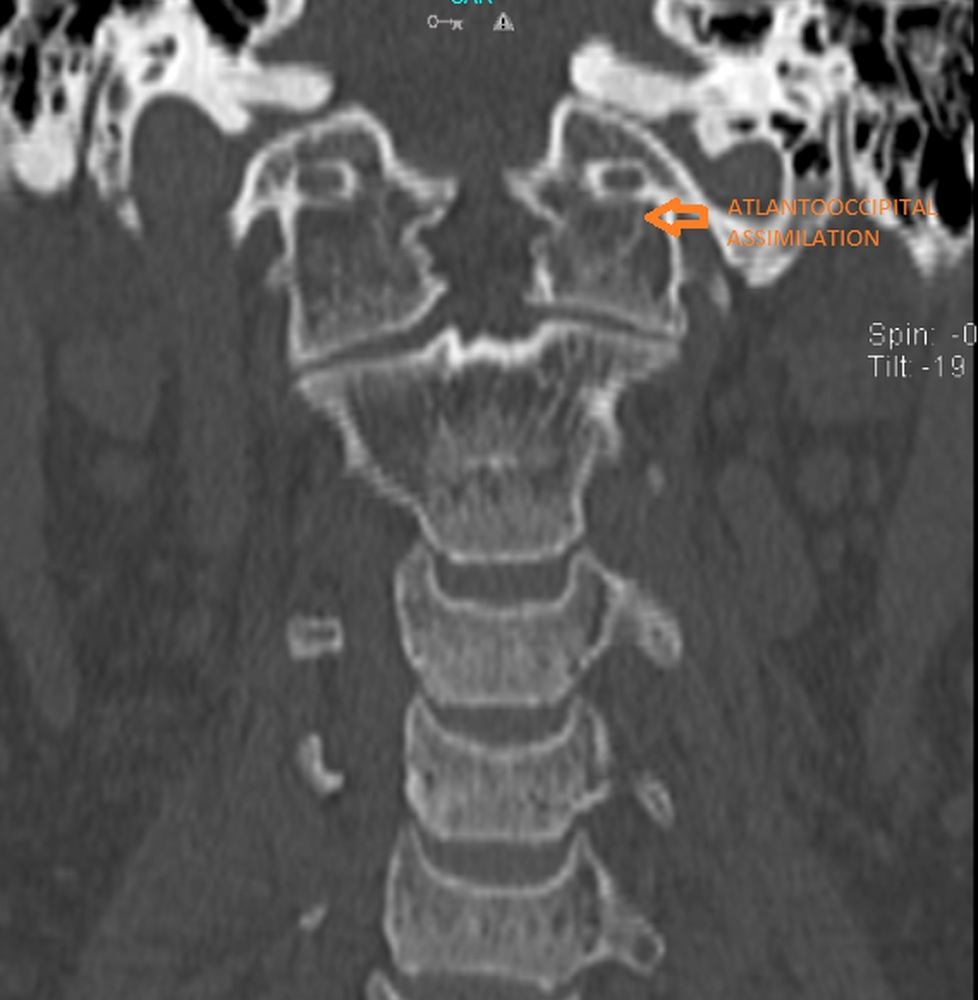
Coronal computed tomogram scan showing complete atlantooccipital assimilation (arrow)

This prompted further medical imaging. A multiplanar, multiecho magnetic resonance imaging (MRI) scan and a computed tomography (CT) scan with 3D reconstruction were performed. The imaging revealed complete atlantooccipital assimilation - complete fusion of the lateral masses of the atlas with the occipital condyles (Figures 2-4).

**Figure 5:**
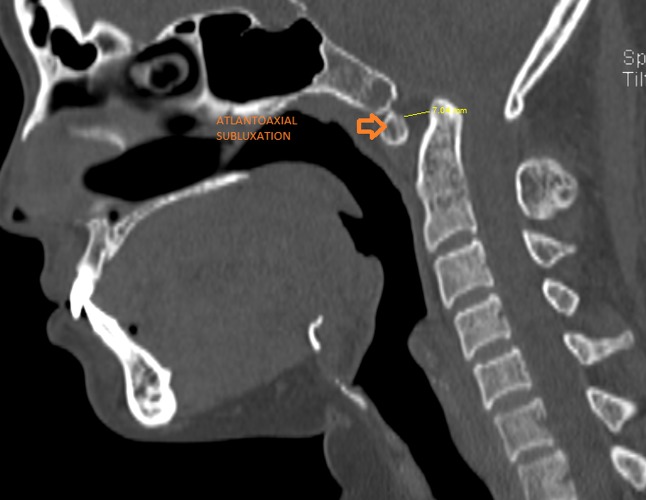
Sagittal computed tomogram showing increased atlanto-dens interval (7 mm) suggestive of atlanto-axial instability (arrow)

A sagittal CT revealed an increased anterior atlanto-dens interval measuring 7 mm suggestive of atlantoaxial instability (Figure 5).

**Figure 6:**
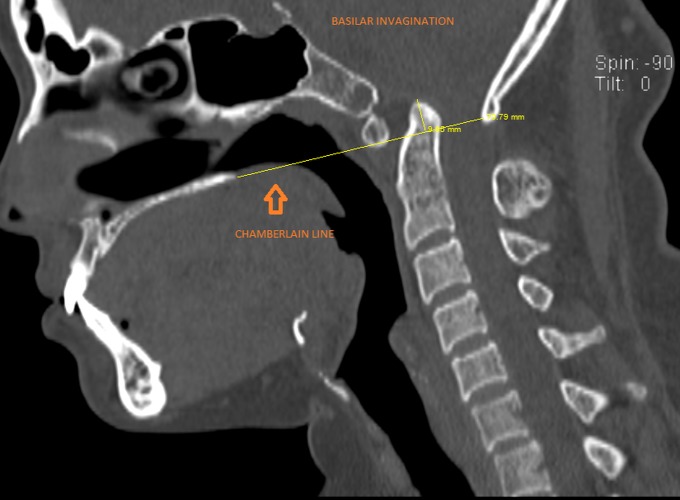
Sagittal computed tomogram showing basilar invagination (tip of the odontoid is located above the Chamberlain line)

The odontoid process of the axis and the anterior arch of the atlas were located above the Chamberlain line, drawn between the posterior pole of the hard palate and opisthion, suggestive of basilar invagination (Figure 6).

**Figure 7:**
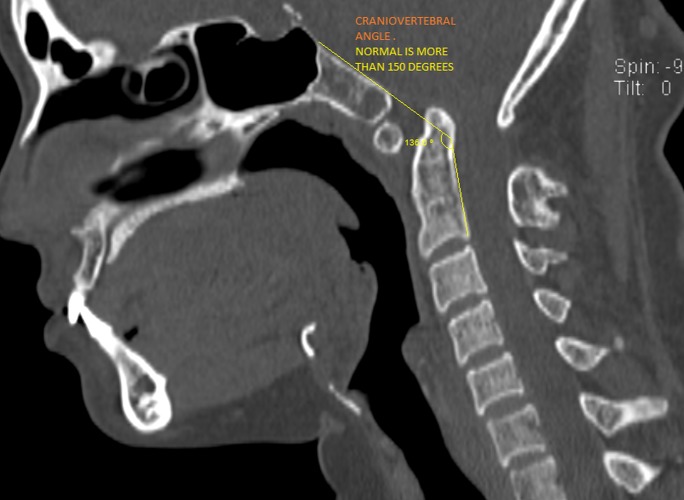
Sagittal computed tomogram showing reduced cranio-vertebral angle

**Figure 8:**
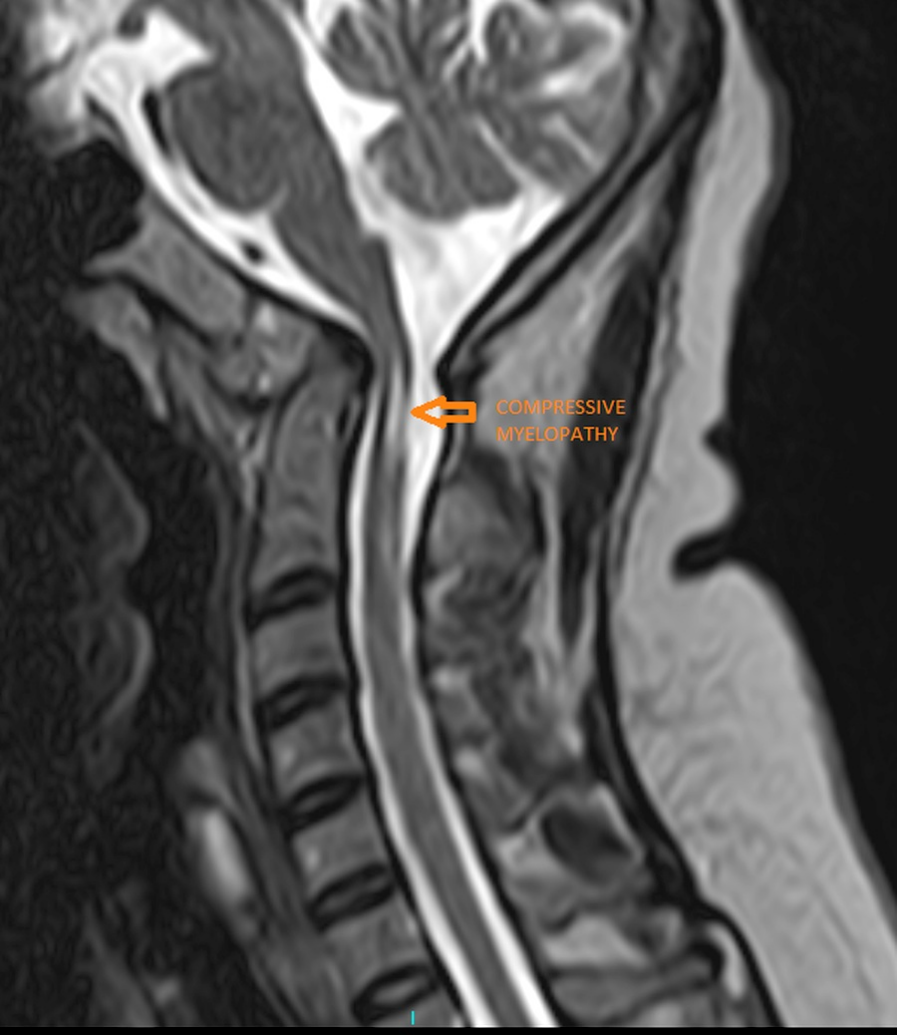
Sagittal T2-weighted magnetic resonance imaging showing compressive myelopathy

**Figure 9:**
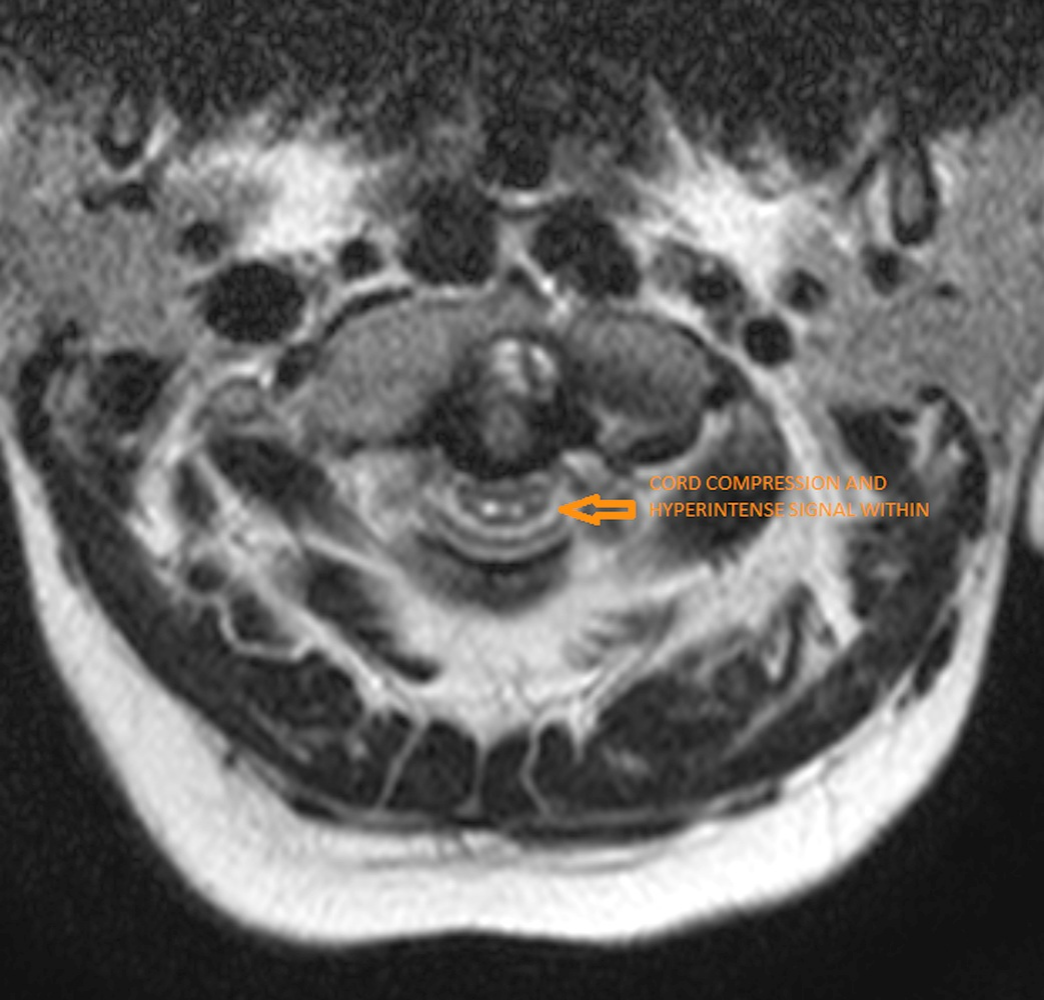
Axial T2-weighted magnetic resonance imaging showing compressive myelopathy

The Welcher basal angle was normal measuring 134 degrees. There was a reduction in the craniovertebral angle (136 degrees), suggestive of predisposition to anterior spinal cord compression, which was seen as a hyperintense signal intensity in the T2 weighted sagittal and axial MRI at the cervicomedullary junction, at the lower border of the C2 vertebra (Figures 7-9).

Since signs of cervical myelopathy and cord compression were absent, the patient was offered non-surgical management with medications, cervical interferential therapy, and a hard cervical collar, Miami- J Collar (Jerome Medical, Moorestown, New Jersey). All symptoms resolved within 10 days of treatment. At eight months follow-up, the patient continues to remain asymptomatic and uses the hard cervical collar most of the time, as advised.

## Discussion

Atlas assimilation or occipitalisation is a partial or complete congenital fusion between the atlas and the occipital bone, ranging from a complete bony fusion to a bony bridge, or a fibrous band uniting one small area of the atlas to the base of the occiput [2]. The incidence of occipitalisation has been reported to be between 0.08% to 3% of the general population, affecting males and females equally [5]. Symptoms usually appear in the third and fourth decades of life. These include neck pain, headache, numbness and pain in the upper extremities, weakness, and symptoms connected to the anterior or posterior spinal cord impingement or vertebral artery compression. McRae and Barnum proposed that the shape of the odontoid is the key to the development of neurological symptoms [6]. Occipitalisation can also lead to chronic atlantoaxial instability and basilar invagination. Patients with this condition often have low hairlines, short necks, and restricted neck movements [7-8]. Since this anomaly ranges from complete assimilation of the atlas into the occiput to a partial connection with a small fibrous band, routine radiographs are usually difficult to interpret. Tomograms, CT scans, or MRI scans may be needed to show the occipitocervical fusion [9]. Myelography can detect areas of spinal cord or medulla compression and is especially useful when a constricting fibrous band is present posteriorly. Patients presenting with minor symptoms or who become symptomatic after a minor trauma, heavy travel, or physical exertion may be treated non-surgically. If neurological symptoms occur, surgical decompression may be necessary. When radiological evidence of atlantoaxial instability is concomitantly present, surgical decompression with occipitocervical fusion has been widely recommended. However, the results of surgical intervention have been variable [10]. In this case, our patient, diagnosed with occipitalisation associated with atlantoaxial instability and basilar invagination without signs of spinal cord compression, responded very well to non-operative treatments in the form of medications, cervical interferential therapy, and a rigid cervical orthosis. The patient uses a hard cervical collar during most of the day as advised. She continues to be asymptomatic at eight months follow-up. The patient has been informed about the need for a future surgical intervention, should she develop symptoms and signs of spinal cord compression.

## Conclusions

We propose that all patients with atlantooccipital assimilation (with or without radiological evidence of basilar invagination and atlantoaxial instability), who do not present with signs of cervical cord compression, must be offered an unbiased non-operative treatment plan before considering surgical intervention. 
